# Limitations to Estimating Mutual Information in Large Neural Populations

**DOI:** 10.3390/e22040490

**Published:** 2020-04-24

**Authors:** Jan Mölter, Geoffrey J. Goodhill

**Affiliations:** Queensland Brain Institute & School of Mathematics and Physics, The University of Queensland, St. Lucia, QLD 4072, Australia; jan.moelter@uq.net.au

**Keywords:** sensory coding, information theory, entropy, sampling bias

## Abstract

Information theory provides a powerful framework to analyse the representation of sensory stimuli in neural population activity. However, estimating the quantities involved such as entropy and mutual information from finite samples is notoriously hard and any direct estimate is known to be heavily biased. This is especially true when considering large neural populations. We study a simple model of sensory processing and show through a combinatorial argument that, with high probability, for large neural populations any finite number of samples of neural activity in response to a set of stimuli is mutually distinct. As a consequence, the mutual information when estimated directly from empirical histograms will be equal to the stimulus entropy. Importantly, this is the case irrespective of the precise relation between stimulus and neural activity and corresponds to a maximal bias. This argument is general and applies to any application of information theory, where the state space is large and one relies on empirical histograms. Overall, this work highlights the need for alternative approaches for an information theoretic analysis when dealing with large neural populations.

## 1. Introduction

The neural code is inherently stochastic, so that even when the same stimulus is repeated, we observe a considerable variability in the neural response. Thus, an important question when considering the interplay between sensory stimuli and neural activity, and when trying to understand how information is represented and processed in neural systems, is how much an observer can actually infer about the stimulus from only looking at its representation in the neural activity. This problem can be formulated and quantitatively studied in the general framework of information theory.

With neural information processing happening at the level of populations, any such analysis necessarily has to consider the activity of an entire population [1,2]. The number of neurons which can be recorded simultaneously has increased in recent years, driven by technologies such as calcium fluorescence imaging [3] and high-density electrode arrays [4]. These methods allow recording of population activity from thousands of neurons simultaneously. However, at these scales, information theoretic analyses of neural activity become considerably harder. Critically, any information theoretic analysis depends at some point on the precise knowledge of the joint probability distribution of the states of the stimuli and the neural population.

However, estimating this distribution from data, and thus entropy and mutual information, is notoriously difficult. This problem is well known, in particular, when the state space for the neural activity becomes large, as is the case when considering large neural populations [5,6,7,8,9]. This problem is generally addressed by applying corrections derived from asymptotic considerations [10], or shuffling/bootstrap procedures together with extrapolations [11,12,13,14,15,16,17]. A crucial result in this context is the inconsistency theorem, which implies that under some circumstances estimators that are frequently employed will yield arbitrarily poor estimates [18]. More recently, and, in particular, with regard to entropy estimation, there have been advances towards new estimators or model-based approaches for estimating the aforementioned joint probability distributions [19,20,21,22,23,24].

In the following we revisit the direct (and model-independent) estimate of entropy and mutual information from experimental histograms. Rhee et al. [7] noted that estimated probability distributions from data samples appear “thinner” and that this leads to what has become known as the sampling bias. Moreover, according to these authors, ensuring the state space is adequately sampled becomes proportionally more difficult with the size of the neural population. The aim of this article is to make this intuition precise, and to show that under certain assumptions with increasing population size any such direct estimate of mutual information will with high probability only yield the stimulus entropy. This emphasises the problems of an information theoretic analysis on the basis of experimental histograms and highlights the importance of new approaches when it comes to estimating information theoretic quatities for large neural populations.

## 2. Results

In a typical experiment investigating sensory processing, one studies the activity from a neural population of interest in response to a defined set of stimuli. Due to the stochastic nature of neural activity, every stimulus is presented separately multiple times, yielding a finite set of samples of neural activity for every stimulus [25]. From these samples across all stimuli, one can then estimate the relative frequency of patterns of neural activity in response to different stimuli in order to, for example, calculate the dependence in terms of mutual information between the stimuli and the neural activity [26,27].

To make this paradigm more concrete, suppose we are interested in the representation of the visual field in the primary visual cortex. In order to probe this representation, we simultaneously record the spiking activity from a neural population in the respective brain area when exposed to a visual stimulus whose position we vary in the course of the experiment. We can sample the neural activity for every presentation of the stimulus, by measuring the neural activity, for example, in terms of the number of spikes. In order then to quantify the representation of the different stimulus values in their entirety, we can collect the samples of neural activity that we observed, compute empirical histograms and perform an information theoretic analysis.

The central objects of interest in information theory are random variables and their distributions. At its core one defines a measure of their uncertainty, the entropy [28]. If *X* is a discrete random variable taking values in an at most countable set, its entropy H(X) is defined as

$$H\left(X\right):=-\sum_{x}P\left[X=x\right]\log P\left[X=x\right],$$

where $P\left[X=\;\cdot \;\right]$ denotes the probability distribution of *X*, and therefore $P\left[X=x\right]$ the probability the *X* attains the value *x*. In this definition, we adopt the customary convention that 0log0:=0. For the purpose of this paper, we leave the base of the logarithm unspecified; however, we note that in the context of information theory the base is generally chosen to be 2 so that entropy will be measured in units of bits.

If $X^{\prime }$ is a second random variable, the conditional entropy of *X* given $X^{\prime }$, $H\left(X|X^{\prime }\right)$, is defined as

H(X|X′):=−∑x,x′PX=x∧X′=x′logPX=xX′=x′X′=x′,

where $P\left[X=\;\cdot \;\wedge X^{\prime }=\;\cdot \;\right]$ and PX=·X′=·X′=· denote the joint probability distribution of *X* and $X^{\prime }$ and conditional probability distribution of *X* given $X^{\prime }$, respectively. The conditional entropy is a measure for the residual uncertainty in a random variable given observation of another random variable. Specifically, we have that $H\left(X|X^{\prime }\right)\leq H(X)$ with equality if *X* and $X^{\prime }$ are independent.

An important quantity in information theory is the mutual information between two random variables, *X* and $X^{\prime }$, which is defined as

$$\mathrm{MI}\left(X;X^{\prime }\right)=H(X)-H\left(X|X^{\prime }\right).$$

Mutual information quantifies the amount of entropy of *X* that knowledge of $X^{\prime }$ annihilates or, in other words, the information that $X^{\prime }$ holds about *X*. Furthermore, as the mutual information is symmetric in its arguments it likewise also quantifies the information *X* holds about $X^{\prime }$. Mutual information is non-negative and vanishes if and only if *X* and $X^{\prime }$ are independent. Because of this, mutual information is frequently used as a measure of the independence of two random variables. The mutual information between *X* and $X^{\prime }$ can be written in terms of the relative entropy (Kullback–Leibler divergence) between their joint probability distributions and the product of the corresponding marginal distributions, which defines a premetric on the probability distribution.

Importantly, we note that, in contrast to what the notation suggests, both the entropy and the mutual information do not depend on the random variables and their values themselves, but are rather functionals of their distributions [29]. As a consequence of that, we refrain from specifying the random variables’ codomains in the following and if a random variable *X* attains the value *x*, *x* may simply be regarded as a label in an abstract alphabet.

### 2.1. Analysis of a Computational Model of Sensory Processing Regarding Estimating Information Theoretic Quantities

**Figure 1:**
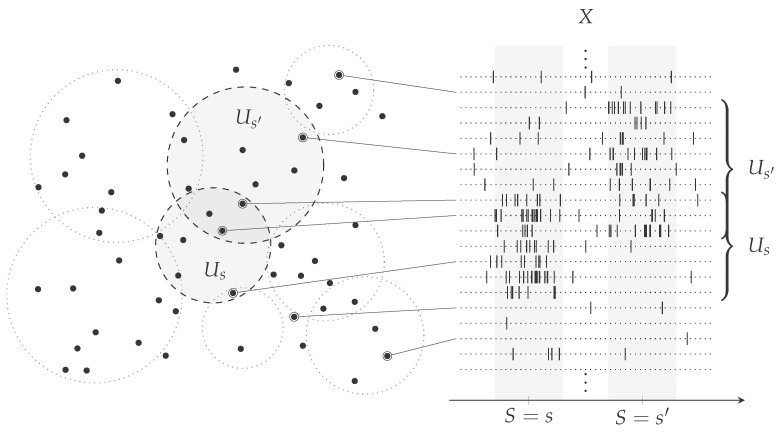
A computational model of sensory processing. We consider a population of *N* neurons, illustrated irrespective of the physical location as points in the plane on the left-hand side that is exposed to the presentation of a stimulus, which is modelled by a random variable *S*. The (measured) activity of this population, illustrated as spiking activity on the right-hand side over the presentation of two different values of the stimulus, is modelled by a vector-valued random variable $X\equiv {⨂}_{n=1}^{N}X^{\left(n\right)}$. For every stimulus value *s*, we assume there exists a subset of the population, $U_{s}$, depicted as circular regions on the plane, such that, intuitively, the neurons within this subpopulation are receptive to the particular stimulus value. Conversely, the neurons in the complement of that set in $\left\{1,\ldots N\right\}$, $U_{⫫s}$, are assumed to be not receptive to that stimulus value and to activate independently according to a common noise profile. As an example, the sets for stimulus values *s* and $s^{\prime }$ are highlighted. Neurons from any of the two sets are shown to have an increased spiking activity during the presentation of the corresponding stimulus value. In contrast to this activity, others are shown to be rather sporadically active.

Building on the experimental paradigm we outlined above, we devise and analyse a simple computational model. We assume the stimulus to be modelled by a discrete, almost surely non-constant random variable *S* and the neural population’s activity by the discrete vector-valued random variable $X\equiv {⨂}_{n=1}^{N}X^{\left(n\right)}$, where *N* is the size of the population. As the stimulus is determined by the experimental protocol, we assume perfect knowledge of its statistics. In addition, we assume that for every stimulus value *s* there exist a subset of the whole neural population $U_{⫫s}\subseteq \left\{1,\ldots N\right\}$ such that $N_{s,s^{\prime }}:=\left|U_{⫫s}\cap U_{⫫s^{\prime }}\right|=\omega \ln N$ as N→∞, i.e., $N_{s,s^{\prime }}$ asymptotically grows faster than the logarithm, for all stimulus values *s* and $s^{\prime }$ such that the components $X^{\left(n\right)}\;|\;S\in \left\{s,s^{\prime }\right\}$ for $n\in U_{⫫s}\cap U_{⫫s^{\prime }}$ are independent and identically distributed and almost surely non-constant (Figure 1). In an experimental setting, one might think of $U_{⫫s}$ as the set of neurons that are not receptive to a stimulus value *s* and therefore activated independently according to a common noise profile. Importantly, these sets differ in general from stimulus to stimulus. However for every two stimulus values, they overlap on a sufficiently large common set. Moreover, in contrast to what one might initially think, these sets cannot be excluded since one can imagine a scenario where every neuron in the population is receptive to at least one of the stimulus values and is simultaneously an element of at least one independent set for other stimulus values.

Next, suppose that for every stimulus value *s* we are given $K_{s}$ independent samples from PX=·S=sS=s, which we denoted as $x^{\left(s\right)}:={\left\{x_{k}^{\left(s\right)}\right\}}_{k=1,\ldots K_{s}}$. Based on these samples the empirical estimate for PX=·S=·S=·, P^X=·S=·S=·, is

P^X=xS=sS=s=1Ks∑k=1Ksδx,xks

for any sample *x* and stimulus value *s*. The Kronecker delta here attains the value 1 whenever its arguments coincide, and otherwise vanishes. Again, in the experimental setting, one might think of $x^{\left(s\right)}$ as the samples of neural activity that was recorded in response to a stimulus value *s*.

Consistent with the intuition of Rhee et al. [7], with high probability, the samples for every stimulus value are mutually different, as we are considering larger and larger neural populations and, moreover, these samples even become uniquely associated with one of the stimulus values, i.e., $x^{\left(s\right)}\cap x^{\left(s^{\prime }\right)}=⌀$ for $s\neq s^{\prime }$. This simplifies the empirical estimate for PX=·S=·S=· from above so that, for a sample $x_{k}^{\left(s\right)}$ and a stimulus value $s^{\prime }$, P^X=xksS=s′S=s′=1Ks′δs,s′, using that every sample is uniquely associated with a stimulus value and occured only once for those stimulus value. As we will show, the probability for this event is at least $1-𝒪\left(N^{-\infty }\right)$ as N→∞. Note, that for a sequence ${\left(z_{N}\right)}_{N\in N}$ we say $z_{N}=𝒪\left(N^{-\infty }\right)$ if $z_{N}=𝒪\left(N^{-m}\right)$ for every m≥0, i.e., $\lim_{N\to \infty }N^{m}z_{N}=0$. This not only implies that in the limit of an infinitely large population, the probability is 1, but also makes a statement about the dependence on the size of population. In fact, the probability approaches 1 eventually faster than any polynomial, so that it will be close to 1 even for moderately sized populations.

For any two stimuli there exists by assumption a subset of the components of *X* which is independent and identically distributed when conditioned on either of the two stimuli. As it suffices that the samples differ in these components for them to be mutually different, the probability for this event is a lower bound on the probability that the samples are mutually different. Therefore, the probability for all samples corresponding to stimulus *s* and $s^{\prime }$ to be mutually different is at least $1-𝒪\left(N^{-\infty }\right)$, for *N* large. As we show in the next section, this is the probability that a finite number of independent samples from a random vector of length $N_{s,s^{\prime }}$ with $N_{s,s^{\prime }}=\omega \left(\ln N\right)$ are mutually different, provided the components are independent and identically distributed and almost surely non-constant, which is the case by assumption.

From that the probability that this event occurs for all stimulus values can be estimated to be at least $1-\sum_{s,s^{\prime }}\left(1-\left(1-𝒪(N^{-\infty })\right)\right)=1-𝒪(N^{-\infty })$.

In that event, when all the samples are mutually different, we compute for the entropy and conditional entropy, $\hat{H}(X)$ and $\hat{H}(X|S)$, using the law of total probability to express both in terms of the distribution of *S* and the empirical estimate of the distribution of *X* given *S*, the fact that the latter vanishes away from the samples and that, as we have seen above, for a sample $x_{k}^{\left(s\right)}$ and a stimulus value $s^{\prime }$, it takes the form P^X=xksS=s′S=s′=1Ks′δs,s′,

H^(X)=−∑xP^X=xlogP^X=x=−∑sPS=s∑xP^X=xS=sS=slog∑s′PS=s′P^X=xS=s′S=s′=−∑sPS=s∑k=1KsP^X=xksS=sS=slog∑s′PS=s′P^X=xksS=s′S=s′=−∑sPS=slogPS=s1Ks=H(S)+ElogKS

and

H^(X|S)=−∑s,xP^X=x∧S=slogP^X=xS=sS=s=−∑sPS=s∑xP^X=xS=sS=slogP^X=xS=sS=s=−∑sPS=s∑k=1KsP^X=xksS=sS=slogP^X=xksS=sS=s=−∑sPS=slog1Ks=ElogKS.

Thus, $\hat{\mathrm{MI}}(X;S)=\hat{H}(X)-\hat{H}(X|S)=H(S)$, and in addition we also get from the above that $\hat{H}(X,S)=\hat{H}(X)$ and that $\hat{H}(S|X)=0$. As for mutual information the classical bound MI(X;S)≤min(H(X),H(S)) holds, mutual information is in fact estimated to be maximal.

Importantly, in this analysis we did not make any assumptions about the precise dependence between *S* and *X* apart from the existence of a sufficiently large subpopulation $U_{⫫s}$ for every stimulus value *s*. Therefore, the conclusion holds also if *S* and *X* were, in fact, independent and mutual information should have vanished.

### 2.2. Computing the Probability of a Finite Number of Independent and Identically Distributed Random Variables being Mutually Different

In the previous section, we were interested in the probability that a finite set of independent and identically distributed (discrete) random variables yields mutually different realisations. This probability is trivially 0 by the pigeonhole principle if there are more random variables in any such set than the number of values these random variables attain with non-vanishing probability. However, once the number of values that are attained exceeds the number of random variables this is not the case anymore, and for any arbitrary distribution of the random variable it is not obvious how to obtain the exact probabilities.

We first take a step back and consider generally series of the form

∑n1,…nK∈Nn1≠n2≠…nKan1⋯anK

for sequences ${\left(a_{n}\right)}_{n\in N}$ such that the corresponding series is absolutely convergent. This specifically guarantees that the series in question will also converge. We will show how to evaluate such a series and from a closed-form expression derive the probabilities for any finite set of random variables to yield mutually different realisations. From this it will follow, in particular, that large random vectors yield mutually different values with high probability.

Let ${\left(a_{n}\right)}_{n\in N}$ be a sequence with $a_{n}\in R$ for every n∈N such that $\sum_{n\in N}a_{n}$ is absolutely convergent and define $Q_{m}:=\sum_{n\in N}a_{n}^{m}$ for m∈N. Then, for any K∈N we have that

∑n1,…nK∈Nn1≠n2≠…nK∏k=1Kank=∑α∈N0K:〈α〉K=K−1K−|α|K!α!∏m=1KQmmαm.

Here and in the following we use the conventional notation for multi-indices $\alpha \in N_{0}^{K}$, so that, e.g., $\left|\alpha \right|=\sum_{k=1}^{K}\alpha_{k}$ and $\alpha !=\prod_{k=1}^{K}\alpha_{k}!$. In addition, we set ${\langle \alpha \rangle }_{k}=\sum_{m=1}^{k}\alpha_{m}m$ for 1≤k≤K.

We will prove this assertion in two steps. We will first derive a recursion relation for the series in question and then in a second step conclude with an induction argument.

We consider a family of operators $\Gamma_{1},\ldots \Gamma_{K}$, and for 1≤k≤K, formally define the corresponding operator $\Gamma_{k}$ on monomials of $a_{n_{k}}$ via

$$\Gamma_{k}:a_{n_{k}}^{m}\mapsto \sum_{n_{k}\in N\backslash \left\{n_{1},\ldots n_{k-1}\right\}}a_{n_{k}}a_{n_{k}}^{m}=Q_{m+1}-\sum_{r=1}^{k-1}a_{n_{r}}^{m+1}$$

and extend it linearly to $\mathrm{span}\left\{1,a_{n_{k}},a_{n_{k}}^{2},\ldots \right\}$. Using these operators we find that

AK:=∑n1,…nK∈Nn1≠n2≠…nK∏k=1Kank=∑n1∈Nan1∑nk∈N\{n1}an2⋯∑nK∈N\{n1,…nK−1}anK=Γ1∘Γ2∘⋯ΓK(1).

In particular, we have for every $1\leq k^{\prime }\leq k\leq K$ and $m\in N_{0}$ that Γ1∘Γ2∘⋯Γk(ank′m)=∑n1,…nk∈Nn1≠n2≠…nkan1⋯ank′m+1⋯ank=∑n1,…nk∈Nn1≠n2≠…nkan1⋯ankm+1=Γ1∘Γ2∘⋯Γk(ankm), where we have relabeled the indices and the interchangability of the sums is guaranteed by the absolute convergence of every individual series. Therefore, $\left(\Gamma_{1}\circ \Gamma_{2}\circ \cdots \Gamma_{k}\right)\left(a_{n_{k^{\prime }}}^{m}\right)=\left(\Gamma_{1}\circ \Gamma_{2}\circ \cdots \Gamma_{k}\right)\left(a_{n_{k}}^{m}\right)$.

Now, using the linearity of the operators $\Gamma_{1},\ldots \Gamma_{K}$ we have that

$$\begin{array}{cc} \left(\Gamma_{1}\circ \Gamma_{2}\circ \cdots \Gamma_{K}\right)\left(a_{n_{K}}^{m}\right) & =\left(\Gamma_{1}\circ \Gamma_{2}\circ \cdots \Gamma_{K-1}\right)\left(Q_{m+1}-\sum_{r=1}^{K-1}a_{n_{r}}^{m+1}\right)\\ \; & =Q_{m+1}\left(\Gamma_{1}\circ \Gamma_{2}\circ \cdots \Gamma_{K-1}\right)\left(1\right)-\sum_{r=1}^{K-1}\left(\Gamma_{1}\circ \Gamma_{2}\circ \cdots \Gamma_{K-1}\right)\left(a_{n_{r}}^{m+1}\right)\\ \; & =Q_{m+1}A_{K-1}-\left(K-1\right)\left(\Gamma_{1}\circ \Gamma_{2}\circ \cdots \Gamma_{K-1}\right)\left(a_{n_{K-1}}^{m+1}\right)\\ \; & =\sum_{k=1}^{K}{\left(-1\right)}^{k+1}\frac{\left(K-1\right)!}{\left(K-k\right)!}Q_{m+k}A_{K-k}. \end{array}$$

In the last step we explicitly resolved the recursion, which can be verified by a simple inductive argument. In particular, for m=0 we obtain an expression for $A_{K}$ so that we conclude after reordering the sum and rearranging the terms that

$$\frac{A_{K}}{{\left(-1\right)}^{K}K!}=-\frac{1}{K}\sum_{k=0}^{K-1}Q_{K-k}\frac{A_{k}}{{\left(-1\right)}^{k}k!}.$$

As $A_{K}$ is precisely the sum we intend to compute, the last expression yields a recursion relation for it with initial datum $A_{0}=1$. In particular, we note that this equation is reminiscent of a discrete Volterra equation of convolution type. Thus, this concludes the first step of the argument and in the second step we will show that the expression stated in the beginning actually solves this recursion relation.

For the induction argument we assume now that the assertion holds for 1≤k≤K−1 in order to perform the step K−1→K and we use the recursion relation derived above to relate $A_{K}$ to $A_{0},A_{1},\ldots A_{K-1}$. In addition, we denote with $e_{k}$ the multi-index that is 0 in every component except the *k*th one, where it is 1.

$$\begin{array}{cc} \frac{A_{K}}{{\left(-1\right)}^{K}K!} & =-\frac{1}{K}\sum_{k=0}^{K-1}Q_{K-k}\frac{1}{{\left(-1\right)}^{k}k!}\left\{\sum_{\alpha \in N_{0}^{k}:{\langle \alpha \rangle }_{k}=k}{\left(-1\right)}^{k-|\alpha |}\frac{k!}{\alpha !}\prod_{m=1}^{k}{\left(\frac{Q_{m}}{m}\right)}^{\alpha_{m}}\right\}\\ \; & =-\frac{1}{K}\sum_{k=0}^{K-1}\sum_{\alpha \in N_{0}^{K}:{\langle \alpha \rangle }_{K}=k}\frac{{\left(-1\right)}^{-|\alpha |}}{\alpha !}Q_{K-k}\prod_{m=1}^{K}{\left(\frac{Q_{m}}{m}\right)}^{\alpha_{m}}\\ \; & =-\frac{1}{K}\sum_{k=1}^{K}\sum_{\alpha \in N_{0}^{K}:{\langle \alpha \rangle }_{K}=K-k}\frac{{\left(-1\right)}^{-|\alpha |}}{\alpha !}Q_{k}\prod_{m=1}^{K}{\left(\frac{Q_{m}}{m}\right)}^{\alpha_{m}}\\ \; & =\sum_{k=1}^{K}\sum_{\alpha \in N_{0}^{K}:{\langle \alpha \rangle }_{K}=K-k}\frac{\left(\alpha_{k}+1\right)k}{K}\frac{{\left(-1\right)}^{-|\alpha +e_{k}|}}{\left(\alpha +e_{k}\right)!}\prod_{m=1}^{K}{\left(\frac{Q_{m}}{m}\right)}^{{\left(\alpha +e_{k}\right)}_{m}}\\ \; & =\sum_{\alpha \in N_{0}^{K}:{\langle \alpha \rangle }_{K}=K}\frac{{\left(-1\right)}^{-|\alpha |}}{\alpha !}\prod_{m=1}^{K}{\left(\frac{Q_{m}}{m}\right)}^{\alpha_{m}} \end{array}$$

In the last step, we used that, as we will show, for any multi-index functional Φ,

$$\sum_{k=1}^{K}\sum_{\alpha \in N_{0}^{K}:{\langle \alpha \rangle }_{K}=K-k}\frac{\left(\alpha_{k}+1\right)k}{K}\Phi \alpha +e_{k}=\sum_{\alpha \in N_{0}^{K}:{\langle \alpha \rangle }_{K}=K}\Phi \alpha .$$

Indeed, we first observe that the terms of Φ that appear in both sums are identical. Therefore, we only have to carefully evaluate the coefficients.

$$\begin{array}{cc} \; & \sum_{k=1}^{K}\sum_{\alpha \in N_{0}^{K}:{\langle \alpha \rangle }_{K}=K-k}\frac{\left(\alpha_{k}+1\right)k}{K}\Phi \alpha +e_{k}\\ \; & =\sum_{\alpha \in N_{0}^{K}:{\langle \alpha \rangle }_{K}=K}\sum_{k=1}^{K}\sum_{\alpha^{\prime }\in N_{0}^{K}:{\langle \alpha^{\prime }\rangle }_{K}=K-k}\frac{\left(\alpha_{k}^{\prime }+1\right)k}{K}\Phi \alpha^{\prime }+e_{k}\delta_{\alpha ,\alpha^{\prime }+e_{k}}\\ \; & =\sum_{\alpha \in N_{0}^{K}:{\langle \alpha \rangle }_{K}=K}\Phi \alpha \sum_{k=1}^{K}\frac{\alpha_{k}k}{K}\sum_{\alpha^{\prime }\in N_{0}^{K}:{\langle \alpha^{\prime }\rangle }_{K}=K-k}\delta_{\alpha ,\alpha^{\prime }+e_{k}}\\ \; & =\sum_{\alpha \in N_{0}^{K}:{\langle \alpha \rangle }_{K}=K}\Phi \alpha \sum_{k=1}^{K}\frac{\alpha_{k}k}{K}\\ \; & =\sum_{\alpha \in N_{0}^{K}:{\langle \alpha \rangle }_{K}=K}\Phi \alpha \end{array}$$

This concludes the argument, so that as claimed

∑n1,…nK∈Nn1≠n2≠…nK∏k=1Kank=∑α∈N0K:〈α〉K=K−1K−|α|K!α!∏m=1KQmmαm.

In order to apply this result now in a probabilistic context to answer the question about the probability that any finite set of (discrete) random variables yields mutually different realisations, consider a discrete random variable *X* that takes values in the set $\left\{x_{1},x_{2},\ldots \right\}$, which we assume without loss of generality to be countably infinite. Then, applying the above result to the sequence ${\left(P\left[X=x_{n}\right]\right)}_{n\in N}$ we can compute the probability that *K* independent copies of this random variable, $X_{1},\ldots X_{K}$, all yield mutually different realisations. Specifically, we have that

$$\begin{array}{cc} P\left[X_{1}\neq X_{2}\neq \cdots X_{K}\right]=E\left[{𝟙}_{X_{1}\neq X_{2}\neq \cdots X_{K}}\right] & =\sum_{x_{1}\neq x_{2}\neq \cdots x_{K}}\prod_{k=1}^{K}P\left[X=x_{k}\right]\\ \; & =\sum_{\alpha \in N_{0}^{K}:{\langle \alpha \rangle }_{K}=K}{\left(-1\right)}^{K-|\alpha |}\frac{K!}{\alpha !}\prod_{m=1}^{K}{\left(\frac{Q_{m}}{m}\right)}^{\alpha_{m}}. \end{array}$$

with $Q_{m}=\sum_{n\in N}P{\left[X=x_{n}\right]}^{m}$.

Now, consider random vectors of increasing length, $X\equiv {⨂}_{n=1}^{f\left(N\right)}X^{\left(n\right)}$, with independent components and assume that there exists ϵ>0 such that $Q_{2}^{\left(n\right)}=\sum_{x}P{\left[X^{\left(n\right)}=x\right]}^{2}\leq 1-\epsilon$ for every n∈N. For the latter a necessary condition is that $X^{\left(n\right)}$ is almost surely non-constant and in particular it is satisfied if the components are identically distributed and almost surely non-constant. Then, if $f\left(N\right)=\omega \left(\ln,N\right)$ increasing, we will show that this implies that in the limit N→∞

$$P\left[X_{1}\neq X_{2}\neq \cdots X_{K}\right]=1-𝒪\left(N^{-\infty }\right),$$

where we recall that for some sequence ${\left(z_{N}\right)}_{N\in N}$ we have that $z_{N}=𝒪\left(N^{-\infty }\right)$ if for every m≥0$\lim_{N\to \infty }N^{m}z_{N}=0$.

Indeed, $0<Q_{m}=\prod_{n=1}^{f\left(N\right)}Q_{m}^{\left(n\right)}\leq {\left(\sup_{n\in N}Q_{m}^{\left(n\right)}\right)}^{f\left(N\right)}\leq {\left(\sup_{n\in N}Q_{2}^{\left(n\right)}\right)}^{f\left(N\right)}\leq {\left(1-\epsilon \right)}^{f\left(N\right)}$ for every m≥2 so that we conclude that $Q_{m}\leq e^{-|\ln1-\epsilon |f\left(N\right)}=𝒪\left(N^{-\infty }\right)$. This implies the claim since $P\left[X_{1}\neq X_{2}\neq \cdots X_{K}\right]$ is a polynomial in terms of $Q_{1},\ldots Q_{K}$ with the only asymptotically non-vanishing term being $Q_{1}^{K}=1$.

Besides the immediate application to random vectors that we required in the last section, we remark that the expression for the probability of random variables being mutually different can also be used to derive a closed-form expression for the Stirling numbers of the first kind. Briefly, let *X* be a uniform random variable on $\left\{1,\ldots L\right\}$ for some L∈N, so that $Q_{m}=L^{1-m}$ in the above statement. The probability that K≤L independent copies of this random variable are all mutually different is then given by the above expression. On the other hand, this probability can also be computed purely combinatorially to be $\frac{{\left(L\right)}_{K}}{L^{K}}$, where ${\left(\;\cdot \;\right)}_{K}$ denotes the falling factorial [30]. By definition, ${\left(L\right)}_{K}=\sum_{n=0}^{K}s\left(K,n\right)L^{n}$ with $s\left(K,n\right)$ the (signed) Stirling numbers of the first kind. Therefore, comparing the two expressions we find the Stirling numbers to be given as (cf. [31])

$$s\left(K,n\right)={\left(-1\right)}^{K-n}\left\{\begin{array}{cc} \sum_{\alpha \in N_{0}^{K}:{\langle \alpha \rangle }_{K}=K}\delta_{|\alpha |,n}\frac{K!}{\alpha !\prod_{m=1}^{K}m^{\alpha_{m}}} & 1\leq n\leq \;K\\ 0 & \mathrm{otherwise} \end{array}\right..$$

## 3. Discussion

In this work, we have analysed a concrete, but general, model of sensory processing and demonstrated the extent of the sampling bias when directly estimating information theoretic quantities from experimental histograms. We have shown that as we consider larger and larger neural populations, with high probability any estimate of entropy or mutual information will only depend on the stimulus entropy.

We found that the issue lies in the fact that the samples of neural activity in response to the presentation of different stimulus values turn out to be mutually distinct. One way this can happen is through the existence of a subpopulation of neurons that only contributes independent noise to the population’s activity. Importantly, the composition of the subpopulation can be stimulus-dependent, so that it is impossible to exclude this subpopulation from the beginning in the analysis. A plausible origin for these noisy subpopulations are sufficiently localised, compactly supported receptive fields. In a simplified scenario, imagine a neural population, whose neurons are receptive to any one of three stimuli. For each of the stimuli, the neurons receptive to one of the other two stimuli constitute such a noisy subpopulation. Now, as we have mentioned also earlier, none of the three subpopulations can be excluded on the grounds that it contributes only noise across all stimuli, yet their activity lets samples recorded from the population appear more distinctive than they are. While for small neural populations the effect of noisy subpopulations can be counteracted by increasing the number of samples that one considers, this becomes infeasible for even moderately sized populations due to the combinatorially large repertoire of population activity. As we have shown, as we consider larger and larger populations and therefore also larger noisy subpopulations the possibility to sample even one activity pattern twice is exponentially suppressed. In reality, receptive fields are localised, yet at least in computational models their range often extends indefinitely. This is the case, for example, for Gaussian receptive fields and the activity of a neuron then depends strongly on some stimulus values while for others the dependence in vanishingly small. While the assumptions of our model are clearly not met, we argue that for all practical purposes the consequence is the same, although weakened. This is because one will only ever consider a finite number of samples. The smaller the dependence between the stimulus and the activity, the weaker it manifests itself in particular when only considering those finitely many samples, so that corresponding neurons appear essentially independent.

In the model we have proposed, we assumed that for any two stimulus values there exist sufficiently large subpopulations such that their components contribute independent and identically distributed noise when exposed to either of the two stimulus values. Now, this is certainly a simplification and a more realistic assumption would be that in the absence of sensory stimulation the activity in this subpopulations is governed by low-dimensional dynamics in addition to some individual noise [32,33]. In principle though, our conclusions should hold true irrespectively following the same general argument that we formalised in this work: In a large neural population, stochastic variability, even if it is only in a relatively small subpopulation, is sufficient to produce unique samples of neural activity in an experiment. In turn, this then manifests itself in a maximal bias when estimating mutual information and other information theoretic quantities.

Without any further assumptions on the statistical relation between the different neurons within the population, this work shows that, despite being a powerful framework, in general, information theoretic analyses become essentially intractable when considering larger neural populations, because of the difficulties of accurately estimating the joint probability distribution between activity and stimulus states from experimental histograms. Therefore, this is where modelling approaches come into play. These approaches frequently employ maximum entropy Ising models that were fit initially only to low-order [34] and later also higher-order statistics of the data [35,36,37]. Alternative approaches include the cascaded logistic model which utilises Dirichlet processes at its core [38] or more recently the population tracking model [24]. However, for an information theoretic analysis it is in addition important to be able to compute the involved quantities in an efficient way. While most models include the possibility to sample states, this is not an option for large populations again because of the issues presented in this work. In the context of maximum entropy Ising models, thermodynamic considerations turned out to be useful to efficiently compute quantities such as the entropy [37,39,40]. In the population tracking model, on the other hand, one derives a reduced, low-dimensional model, from which those quantities can be computed likewise [24].

Altogether, while for many statistics of neural activity it might be sufficient to consider experimental histograms, this is not the case for information theoretic quantities when considering large neural populations. Given the many insights that information theoretic analyses can bring [41,42,43], this motivates new approaches for studying experimental recordings from ever larger neural populations. Furthermore, it also inspires consideration of how the brain handles the informational constraints we have identified.
